# Long-Term Reduction of T-Cell Intracellular Antigens Reveals a Transcriptome Associated with Extracellular Matrix and Cell Adhesion Components

**DOI:** 10.1371/journal.pone.0113141

**Published:** 2014-11-18

**Authors:** Mario Núñez, Carmen Sánchez-Jiménez, José Alcalde, José M. Izquierdo

**Affiliations:** Centro de Biología Molecular ‘Severo Ochoa’, Consejo Superior de Investigaciones Científicas, Universidad Autónoma de Madrid (CSIC/UAM), Madrid, Spain; INSERM, France

## Abstract

Knockdown of T-cell intracellular antigens TIA1 and TIAR contributes to a cellular phenotype characterised by uncontrolled proliferation and tumorigenesis. Massive-scale poly(A+) RNA sequencing of TIA1 or TIAR-knocked down HeLa cells reveals transcriptome signatures comprising genes and functional categories potentially able to modulate several aspects of membrane dynamics associated with extracellular matrix and focal/cell adhesion events. The transcriptomic heterogeneity is the result of differentially expressed genes and RNA isoforms generated by alternative splicing and/or promoter usage. These results suggest a role for TIA proteins in the regulation and/or modulation of cellular homeostasis related to focal/cell adhesion, extracellular matrix and membrane and cytoskeleton dynamics.

## Introduction

T-cell intracellular antigen 1 (TIA1) and TIA1 related/like (TIAR/TIAL1) are two proteins that impact several molecular aspects of RNA metabolism at different transcriptional and post-transcriptional layers of gene expression [1]–[4]. In the nucleus, TIA proteins regulate and/or modulate DNA-dependent transcription by interacting with DNA and RNA polymerase II [5]–[8]. Also, they facilitate splicing of pre-mRNAs (around 10–20% of splicing events in human genome) *via* improving the selection of constitutive and atypical 5′ splice sites through shortening the time available for definition of an exon by enhancing recognition of the 5′ splice sites [9]–[12]. In the cytoplasm, they regulate and/or modulate localization, stability and/or translation of human mRNAs by binding to the 5′ and/or 3′ untranslatable regions [13]–[25]. Therefore, these multifunctional proteins impress prevalently on the molecular and cellular biology of specific RNAs and proteins, altering their lives and destinies in response to environmental cues and challenges.

TIA proteins appear to have a pleiotropic role in the control of cell physiology. For example, they have been shown to play an important role during embryogenesis. Accordingly, mice lacking TIA1 and TIAR die before embryonic day 7, indicating that one or both proteins must be properly expressed for normal early embryonic development. Indeed, mice lacking TIA1 or TIAR, or ectopically over-expressing TIAR, show higher rates of embryonic lethality [17], [26]–[28]. Further, TIA regulators are known to target genes with relevant biological associations with cell networks involving complex responses such as death/survival, proliferation/differentiation, inflammation, adaptation to environmental stress, viral infections and tumorigenesis [1], [2], [13]–[28].

Although the relevance of TIA proteins in key cellular processes involving, for example, inflammation and the stress responses, are well established, their roles on proliferation/differentiation events and survival/cell death responses in patho-physiological settings are not completely known. To assess the potential long-term regulatory roles of TIA proteins in cellular responses, we used an RNA interference strategy to stably down-regulate TIA1/TIAR expression together with genome-wide profiling analysis, to identify genes and processes involved in cell phenotypes regulated and/or modulated by TIA proteins. Our findings suggest that TIA proteins regulate and/or modulate membrane dynamics linked to extracellular matrix and focal/cell adhesion components.

## Materials and Methods

### Cell cultures and immunofluorescence analysis

Adherent HeLa cell lines, silenced for expression of TIA1, TIAR and HuR, or control cells, were constructed by stable transfection of corresponding short hairpin RNAs (shRNAs) (Fig. S1). Cell lines were maintained under standard conditions and analyzed by confocal microscopy [23]–[25].

### RNA purification

Total RNA was purified with TRIzol Reagent (Invitrogen). RNA quality was assessed using the Agilent 2100 Bioanalyzer.

### Library preparation and sequencing

cDNA libraries were prepared with Illumina’s mRNA-Seq Sample Prep kit following the manufacturer’s protocol. Each library was run on one RNASeq Multiplexed 75-bp paired-end sequence using the Illumina Genome Analyzer (GAIIx), facilitated by the Madrid Science Park.

### Primary processing of Illumina RNA-seq reads

RNA-seq reads were obtained using Bustard (Illumina Pipeline version 1.3). Reads were quality-filtered using the standard Illumina process. Three sequence files were generated in FASTQ format; each file corresponded to the HeLa cell line from which the RNA originated [29]. The total number of reads and additional metrical data are shown in Fig. S2. The sequence data have been deposited in the NCBI Gene Expression Omnibus database (http://www.ncbi.nlm.nih.gov/geo/info/linking.html) and are accessible through the GEO Series accession number GSE46516.

### Mapping of RNA-seq reads using TopHat

Reads were processed and aligned to the University of California, Santa Cruz, reference human genome (UCSC, build hg19) using the TopHat tool [30].

### Transcript assembly and abundance estimation using Cufflinks

The aligned read files were processed using the Cufflinks software suite [31]. Cufflinks uses the normalized RNA-seq fragment counts to measure the relative abundance of transcripts. The unit of measurements is fragments per kilobase of exon per million fragments mapped (FPKM). Confidence intervals for FPKM estimates were calculated using Bayesian inference [32].

### Comparison of reference annotation and differential expression testing using Cuffcompare and Cuffdiff

Once all short read sequences were assembled with Cufflinks, the output. GTF files were sent to Cuffcompare along with a reference. GTF annotation file downloaded from the Ensembl database. This classified each transcript as known or novel. Cuffcompare produces a combined. GTF file which is passed to the Cuffdiff tool with the original alignment (.SAM) files produced by TopHat. We used Cuffdiff to perform two pairwise comparisons of expression, splicing and promoter use between control, TIA1 or TIAR-silenced samples.

### Visualization of mapped reads

Mapping results were visualized using both the UCSC genome browser [33], [34] and the Integrative Genomics Viewer software, available at http://www.broadinstitute.org/igv/. Views of individual genes were generated by uploading coverage.wig files to the UCSC Genome browser as a custom track. Data files were restricted to the chromosome in question due to upload limits imposed by the genome browser. The same method was used to generate coverage plots for all chromosomes.

### Genome-wide profiling by microarray analysis

RNA quality checks, amplification, labelling, hybridization with Human Genome U133 Plus 2.0 Array Chips (approximately 55,000 transcripts) (Affymetrix Inc. Santa Clara, CA) and initial data extraction were performed at the Genomic Service Facility at the Centro Nacional de Biotecnología (CNB-CSIC). Comparison of multiple cDNA array images (three independent experiments per each biological condition tested) was carried out by using an average of all of the gene signals on the array (global normalization) to normalize the signal between arrays. The quantified signals were background-corrected (local background subtraction) and normalized using the global Lowess (LOcally WEighted Scatterplot Smoothing) regression algorithm. Local background was corrected by the normexp method with an offset of 50. Background-corrected intensities were transformed to log scale (base 2) and normalized by Lowess for each array [35]. Finally, to obtain similar intensity distribution across all arrays, Lowess-normalized-intensity values were scaled by adjusting their quantiles [36]. After data processing, each probe was tested for changes in expression over replicates using an empirical Bayes moderated *t* statistic [37]. To control the false discovery rate (FDR), *P* values were corrected using the method of Benjamini and Hochberg [38]. FIESTA viewer (http://bioinfogp.cnb.csic.es/tools/ FIESTA) was used to visualize all microarray results and to evaluate the numerical thresholds (–1.5≥ fold change ≥1.5; FDR<0.05) applied for selecting differentially expressed genes [39]. Microarray data have been deposited in the NCBI Gene Expression Omnibus database (http://www.ncbi.nlm.nih.gov/geo/info/linking.html) and are accessible through the GEO Series accession number GSE47664.

### Functional analysis of gene lists

The Gene Ontology (GO) and Kyoto Encyclopedia of Genes and Genomes (KEGG) database analyses were conducted using software programmes provided by GenCodis3 (http://genecodis.cnb.csic.es) [40], [41]. The functional clustering tools were used to look for functional enrichment for significantly over- and under-expressed genes (*P*<0.05) in control, TIA and/or TIAR-silenced HeLa cell lines.

### QPCR analysis

Reverse transcription (RT) reactions and real-time polymerase chain reaction (PCR) was performed at the Genomic PCR Core Facility at Universidad Autónoma de Madrid in the Centre of Molecular Biology *Severo Ochoa*. Analysis was performed on two independent samples in triplicate, including no-template and RT-minus controls. Beta-actin (ACTB) mRNA expression was used as an endogenous reference control. Relative mRNA expression was calculated using the comparative cycle threshold method. The primer pairs used in the analysis are described in Fig. S8. The following mRNAs were quantified: TIA1, T-cell intracellular antigen 1; TIAR, TIA1 related protein; ACTB; beta-actin; CLGN, calmegin; COL1A2, collagen type 1, alpha 2; FAM129A, cell growth inhibiting protein 39; FBN2, fibrillin 2; LGR5, leucine-rich repeat containing G protein-coupled receptor 5; MKX, mohawk homeobox; TWIST1, twist basic helix-loop-helix; AKR1C1-4, aldo-keto reductase family, members C1–C4; CCL2, chemokine (C-C motif) ligand 2; COL4A4, collagen type IV, alpha 4; COL5A3, collagen type V, alpha 3; OSGIN1, oxidative stress induced growth inhibitor 1 and TNFRF11B, tumor necrosis factor receptor superfamily member 11b.

### Fluorescence microscopy

Control and TIA-silenced HeLa cells were grown for 24 h on coverslips, washed with phosphate-buffered saline (PBS), fixed in formalin (Sigma) at room temperature for 10 min, washed with PBS, and processed. For immunofluorescence, the coverslips were incubated for 45 min at room temperature with the primary antibodies against TIA1, TIAR, HuR (Santa Cruz Biotechnology) or α-tubulin (Sigma) proteins [23], [24]. The samples were then washed with PBS and incubated for 45 min with the corresponding secondary antibodies (Invitrogen). The samples were then washed in PBS and mounted with Mowiol (Calbiochem). Microscopy was performed with a confocal microscope.

### Cell adhesion assays

A cell adhesion assay was carried out as described [42]. Briefly, control and TIA-silenced HeLa cells were exponentially grown in DMEM supplemented with 10% FBS (Sigma). Cells were deprived of serum for 12 h prior to the assay and then were washed three times with serum-free DMEM and grown in DMEM. Cells were detached using 10 mM EDTA and observed under a microscope to confirm complete dissociation (aprox. 10 min.). The cells were then washed twice with DMEM to remove EDTA and were resuspended at 2×10^5^ cells/ml in DMEM/0.1% BSA in 12-well plates coated with either BSA (10 µg/ml), rat collagen I (150 µg/ml) or non-coated. Cells (500 µl) were allowed to adhere for 20 min at 37°C. Then, each well was washed (four times) with 300 µl DMEM to eliminate non-adherent cells. After washing, DMEM containing 10% FBS was added and cells were allowed to recover at 37°C for 4 h. Following this, 30 µl of MTT substrate (5 mg/ml) was added to each well and the incubation was continued for an additional 2 h at 37°C. Finally, the MTT-treated cells were lysed in buffer containing 10% SDS and 0.03% HCl and the absorbance was measured at 570 nm on a spectrophotometer. Where indicated, cells were cultured in the presence of DMSO or PMA (100 nM) for 4 h and assayed for MTT activity.

### Transfections and luciferase assays

The human COL1A2 promoter construct containing the partial promoter sequence fused to a firefly luciferase (Luc) reporter gene was generated as previously described [43]. This construct was kindly provided by Prof. Miyazahi (Tokyo Medical University, Tokyo, Japan). Control, TIA1 and/or TIAR-silenced HeLa cells were transiently co-transfected with 500 ng of pEGFP-C1 and firefly luciferase reporter plasmids. Cells were incubated at 37°C for 24 h and lysed with 200 µl of cell culture lysis reagent (Promega) and microcentrifugated at 14,000 rpm for 5 minutes at 4°C; supernatant was used to determine firefly luciferase activity in a Monolight 2010 luminometer (Analytical Luminiscence Laboratory). Luciferase activity was expressed as relative light units (RLU) per milligram of protein and normalized to GFP expression determined by immunoblot. Co-transfection experiments were performed in duplicate and the data presented as the means of the ratio RLU/GFP, expressed as fold induction relative to the corresponding control values (means ± standard error of the mean).

## Results

### RNA-seq coverage

**Figure 1:**
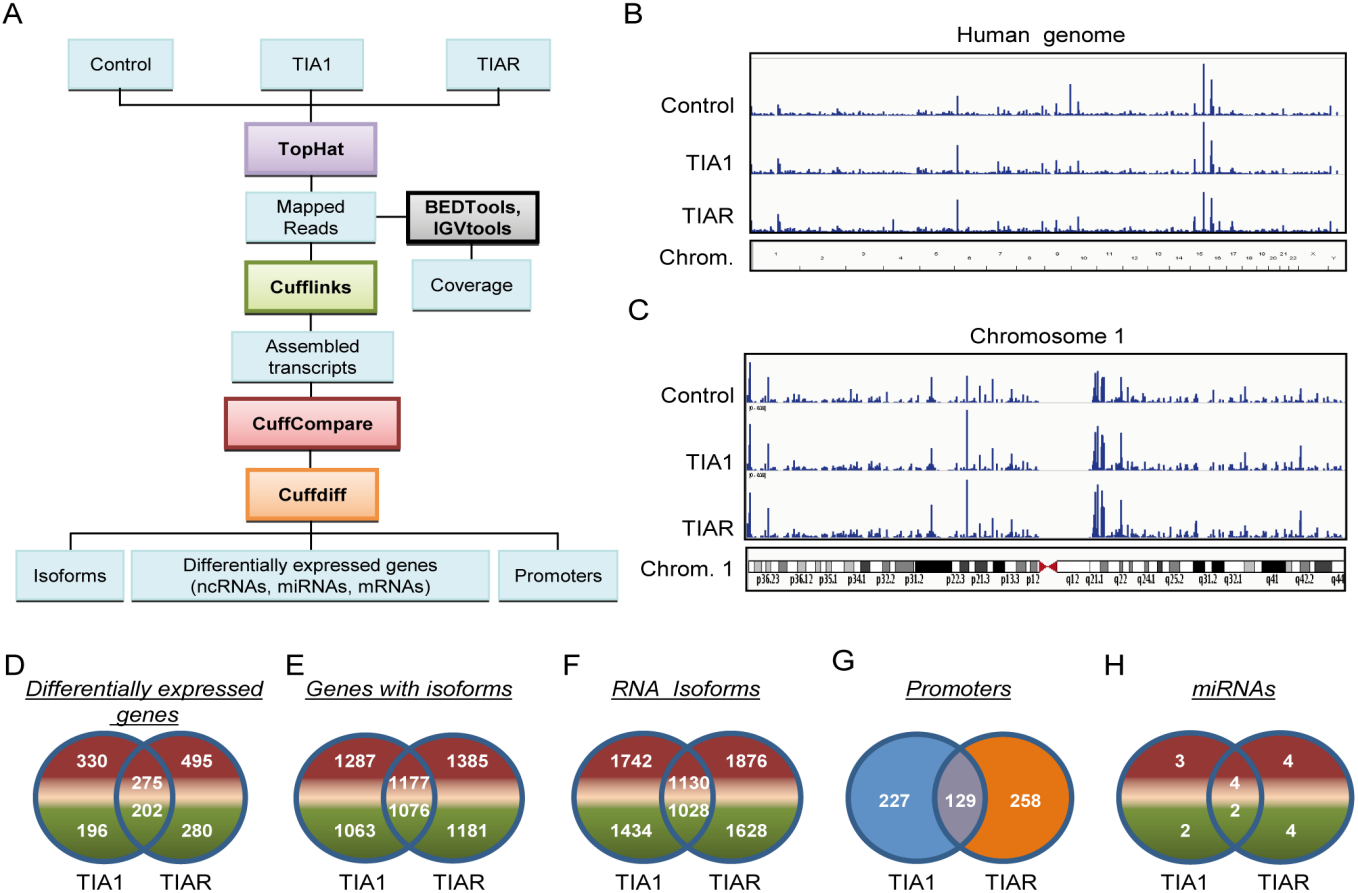
Characterization by massive poly(A^+^) sequencing of the transcriptomes associated with TIA1 or TIAR-silenced HeLa cells. (**A**) Workflow followed to analyze the data from massive-scale poly(A+) RNA sequencing. (**B** and **C**) Transcriptional profiling in human chromosomes. The RNA-seq read density along the length of the human genome (**B**) and chromosome 1 (**C**) are shown. (**D**–**H**) Venn diagrams displaying distributions and numbers of differentially-expressed genes (**D**), genes with isoforms (**E**), RNA isoforms (**F**), promoters (**G**) and microRNAs (**H**) in TIA1 or TIAR-silenced HeLa cells. The numbers of genes that were up- (in red) and down- (in green) regulated, as well as those shared, are indicated.

To study the individual contribution of TIA1 and TIAR proteins on a genome-wide basis, we set out to characterize the transcriptomes associated with TIA1 or TIAR-down-regulation in HeLa cells ([25] and Fig. S1). Three transcriptomic libraries were generated from poly(A+) RNAs isolated from control, TIA1 or TIAR-knocked down HeLa cells, and were analyzed using the Illumina platform. The alignment and annotation of resulting reads, splice junctions and profiles of differentially-expressed RNAs were performed with bioinformatic tools indicated in Fig. 1A [29]. A total of 67.37 M reads were obtained from control, TIA1 and TIAR-silenced cells (Fig. S2). Unique reads were mapped to the *Homo sapiens* genome for each condition. The distribution of mapped reads to different chromosomal features revealed that a significantly large number (46.3%, 46.4% and 47.9%, respectively) of the reads mapped within mRNA coding regions. The remaining reads were mapped within untranslated (UTR) (23.6%, 23.8% and 23.3%, respectively), intronic (11.8%, 11.9% and 11.9%, respectively) and intergenic (18.1%, 17.8% and 16.7%, respectively) regions (Fig. 1B and Fig. S2).

To investigate the level and uniformity of the read coverage against the human genome, we plotted mapped reads of the control, TIA1 and TIAR samples along the human genome as well as for each human chromosome (Fig. S3). The coverage of the RNA-seq analysis on the human genome was extensive, as illustrated by chromosome 1 since this is the largest chromosome in the human karyotype, encoding over 13.6% of all human genes. As expected, no reads mapped to the centromeres but revealed extensive transcriptional activity throughout the chromosomes (Fig. 1B and C and Fig. S3). The distribution of reads within distinct chromosomal regions indicated a very homogeneous scattering among the experimental situations compared (Fig. 1B and C and Fig. S3). Between 91% (corresponding to control sample) and 92% (corresponding to both TIA1 and TIAR samples) of reads aligned to the reference genome in a unique manner. Due to low quality, a small percentage of reads were removed from the analysis prior to mapping to the reference (Fig. S2 and S3).

### Analysis of differentially expressed genes (DEGs), genes with isoforms, RNA isoforms, promoters and microRNAs

Cufflinks uses the Cuffdiff algorithm to calculate differential expression at both the gene and transcript level ([29] and Fig. 1A). Differential gene expression for control versus TIA1 or TIAR samples was calculated using the ratio of TIA1 or TIAR versus control FPKM values for every gene. The differential gene expression ratios were tested for statistical significance as described [29]. To detect transcriptional regulation, RNA-seq data was analyzed by Cufflinks. Cufflinks also identifies post-transcriptional regulation by looking for changes in relative abundances of mRNAs spliced from the same primary transcript between control and TIA1 or TIAR-silenced conditions, which is detected as alternative splicing. Thus, Cufflinks discriminates between transcriptional and post-transcriptional processing ([29] and Fig. 1A). Using Venn diagrams, a summary of the number of shared and differential expressed genes (DEGs), genes with isoforms (splicing data filtered by gene name), RNA isoforms (splicing data filtered by NM identifier), promoters and micro(mi)RNAs is shown in Fig. 1D–H and Fig. S4–S6. Collectively, these results indicate that TIA1 and/or TIAR regulate both specific and overlapping aspects of the human transcriptome, suggesting that their functional roles can be redundant, additive and/or independent, in agreement with previous findings [22], [24], [25], [28], [44].

### Classification and cluster analysis of genes, RNAs and promoters identified by RNA-seq

**Figure 2:**
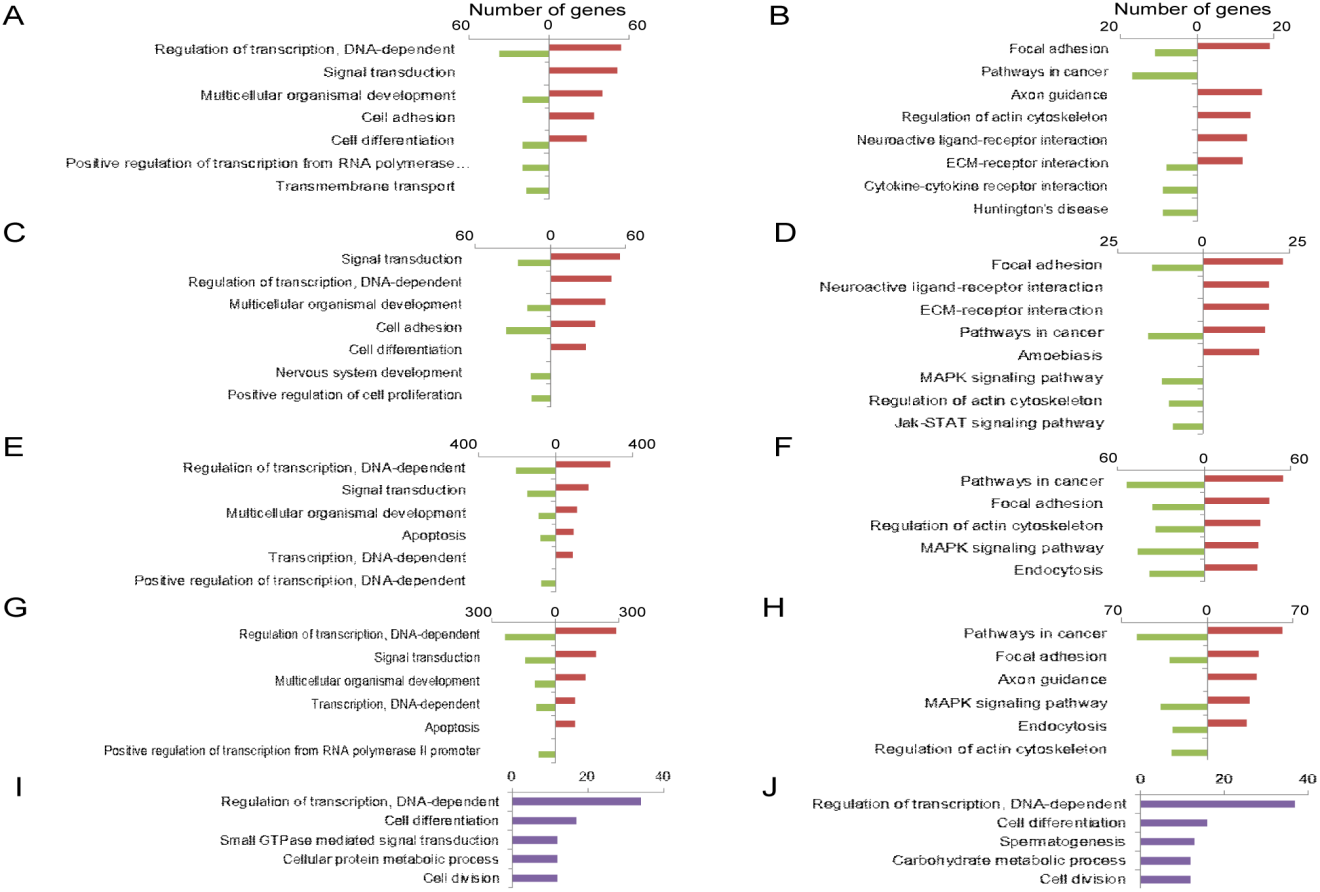
Top-five categories of biological processes and pathways associated with TIA1 or TIAR-silenced HeLa cells. (**A**–**D**) Histograms of the distribution of up- (red) and down- (green) regulated genes using the Gene Ontology (GO) (**A** and **C**) and Kyoto Encyclopedia of Genes and Genomes (KEGG) (**B** and **D**) databases (*P*<0.05) in TIA1 (**A** and **B**) or TIAR (**C** and **D**)-silenced HeLa cells. (**E**–**H**) Histograms of the distribution of up- (red) and down- (green)-regulated RNA isoforms using the GO (**E** and **G**) and KEGG (**F** and **H**) databases (*P*<0.05) in TIA1- (**E** and **F**) or TIAR- (**G** and **H**) silenced HeLa cells. (**I** and **J**) Histograms of the gene distribution of alternative promoter usage in the GO database (*P*<0.05) in TIA1- (**I**) or TIAR- (**J**) silenced HeLa cells.

As a first attempt to understand the functional relevance of differentially expressed up- and down-regulated genes, isoforms and promoters in TIA or TIAR-silenced HeLa cells, Gene Ontology (GO) and Kyoto Encyclopedia of Genes and Genomes (KEGG) database analysis was performed. GO and KEGG analysis were able to identify the main categories of biological processes and pathways of DEGs controlled by TIA1 and TIAR (*P*<0.05). GO categories related to regulation of DNA transcription, signal transduction, multicellular organismal development, cell adhesion and differentiation, cell proliferation, apoptosis and nervous system were among the enriched categories in both up- and down-regulated genes obtained through silencing of TIA1 or TIAR (Fig. 2A, C, E, and G and Fig. S4–S6). KEGG database analysis, integrating individual components into unified pathways, was used to identify the enrichment of specific pathways in functionally-regulated gene and RNA groups. The results showed that several KEGG pathways were significantly enriched (*P*<0.05) in both up- and down-regulated genes, including pathways involved in focal adhesion, cancer, axon guidance, extracellular matrix components (EMC)-receptor interaction, MAPK signalling and regulation of actin cytoskeleton (Fig. 2B, D, F, and H and Fig. S4–S6). GO categories related to alternative promoter usage involved biological processes associated with DNA transcription, cell differentiation and cycle, signal transduction and metabolism (Fig. 2I and J and Fig. S4–S6). Collectively, these results suggest that both TIAR and TIA1 modulate specific and overlapping aspects of the transcriptome (DEGs, alternative splicing and promoters) related to the extracellular environment and signal transduction pathways, which could contribute to the cell proliferation and differentiation phenotypes described in TIA1 and/or TIAR-knocked down HeLa cells [19], [25].

### Microarray analysis and functional categories

**Figure 3:**
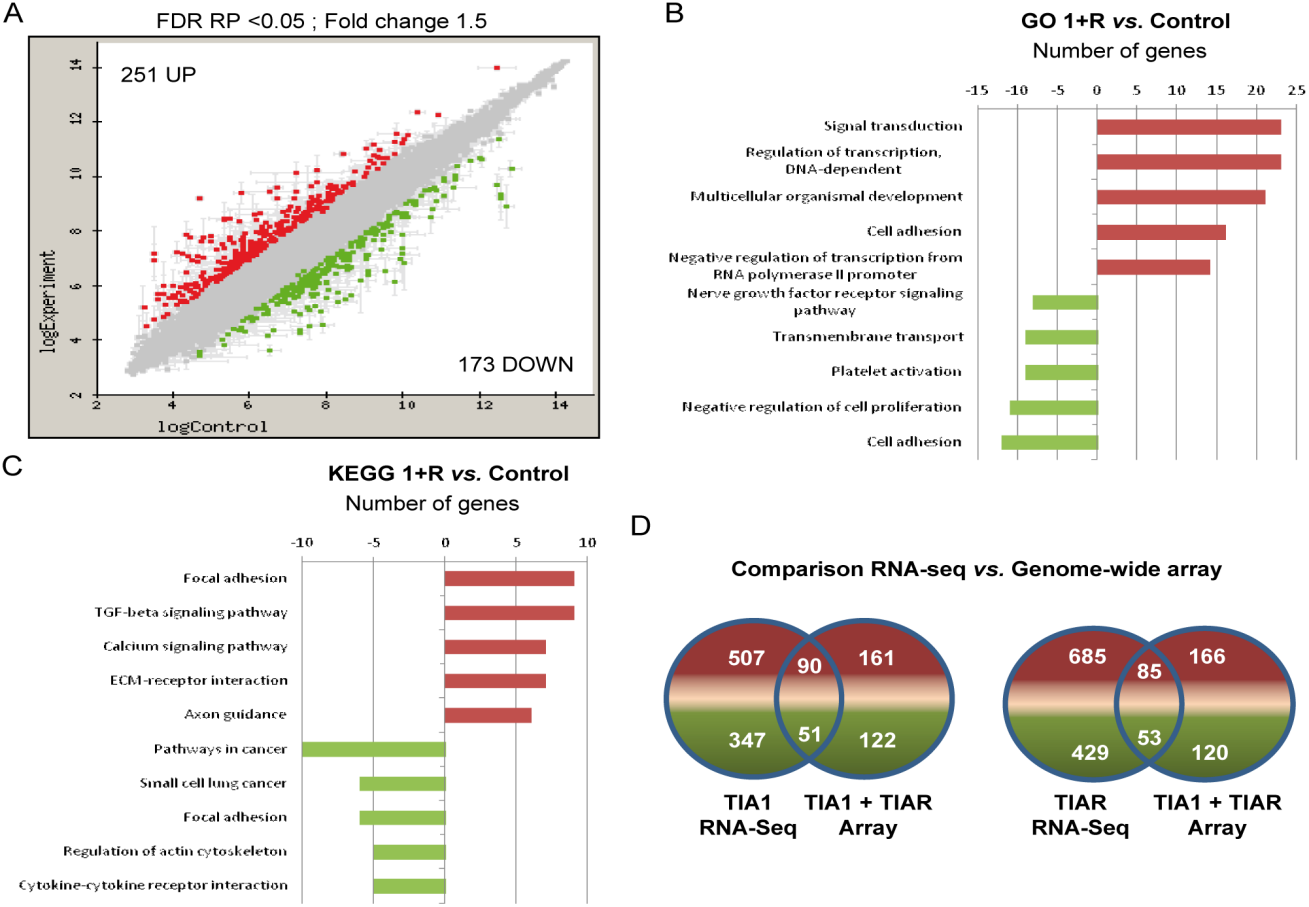
Characterization by genome-wide profiling of the transcriptome associated with TIA-silenced HeLa cells. (**A**) MA plot representation of the distribution of up- (spots in red) and down- (spots in green) regulated RNAs (–1.5≥ fold-change ≤1.5; FDR<0.05) in TIA1 and TIAR-silenced *versus* control HeLa cells. (**B** and **C**) Histograms of the distribution of up- (red) and down- (green) regulated genes using the GO (**B**) and KEGG (**C**) databases (*P*<0.05) in TIA1 and TIAR-silenced HeLa cells. (**D**) Comparison between RNA-seq and genome-wide array data. Venn diagrams depicting the numbers of genes that were up- (red) and down- (green) regulated as well as those shared in TIA1 or/and TIAR-knocked down HeLa cells.

To validate genes and regulatory trends found after silencing both TIA proteins, we used genome-wide expression microarrays. This yielded a set of differentially expressed genes in TIA1 and TIAR-silenced HeLa cells: 251 genes were up-regulated and 173 genes were down-regulated by more than 1.5-fold (FDR RP<0.05) (Fig. 3A and Fig. S7). GO and KEGG database analysis of the up- and down-regulated genes showed significant enrichments in genes associated with signal transduction, cell and focal adhesion, multicellular organismal development and the nervous system (Fig. 3B and C). A comparison of the results obtained from RNA-seq and genome-wide analysis revealed the superior power of massive-RNA sequencing (Fig. 3D), suggesting that our understanding of transcriptional complexity linked to TIA proteins is far from complete.

### Validation of RNA-seq and mRNA array-predicted changes by QPCR

**Figure 4:**
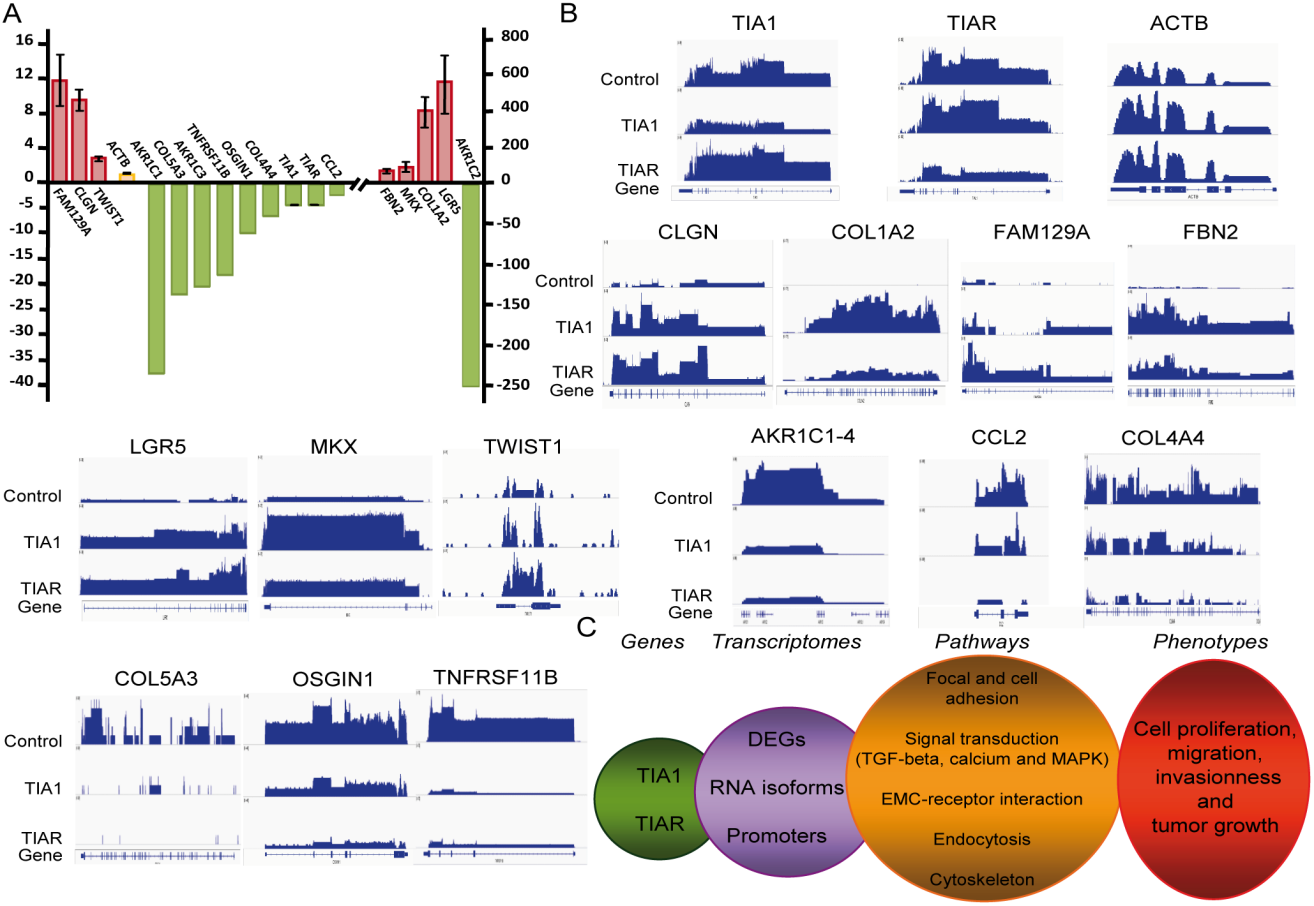
Validation of RNA-seq and microarray predicted changes. (**A**) Quantification of relative expression levels of indicated genes by qPCR. (**B**) RNA-seq mapping to the UCSC reference genome (hg19) of the genes indicated in control and TIA1 or TIAR-knocked down HeLa cells. A schematic representation of every gene analyzed is shown at the bottom. (**C**) Summary of the molecular and cellular events associated with transcriptome dynamics in TIA-downregulated HeLa cells.

To validate previous results on identified genes and their relative expression levels, several up- and down-regulated mRNAs were confirmed by quantitative RT-PCR (QPCR) analysis (Fig. S8). As shown in Fig. 4, the results obtained by quantitative amplification (Fig. 4A) were fully consistent with the data observed by hybridization using microarrays, and the relative fold changes in individual massive RNA sequencing for TIA1 or TIAR (Fig. 4B).

### Knockdown of TIA proteins results in increased cell adhesion rates

As shown in Fig. 2, 3 and S4–S7, GO and KEGG analysis suggested a long-term role for TIA1 proteins in molecular events linked with cell adhesion. To validate these results at the functional level, we examined the cell adhesion potential of HeLa cells with reduced TIA1 and TIAR expression. As shown in Fig. S9, the reduction of TIA1 plus TIAR expression in HeLa cells resulted in an increased cell adhesion capacity compared with control HeLa cells. Indeed, measurement of metabolic activity by methyl thiazolyl tetrazolium (MTT) assay, support a role for TIA proteins in the regulation and/or modulation of cell-substratum adhesion. This capacity, associated with the reduction of the levels of TIA proteins, was stimulated with phorbol 12-myriaste 13-acetate (PMA) in control HeLa cells after 4 hours of incubation (Fig. S9). Furthermore, as shown in Fig. S10, the sustained reduction of TIA1 and/or TIAR could promote a significant and reproducible induction (2- to 16-fold) of a luciferase construct under the control of COL1A2 human promoter sequences [43], suggesting that diminished expression of TIA proteins deregulates the basal transcriptional activity of this promoter. Taken together, these observations may suggest that the gene expression patterns detected in TIA1/TIAR-knocked down HeLa cells might be the result of an overlapping regulation, implying the involvement of several molecular events regulated and/or modulated by these multifunctional proteins on cell adhesion and extracellular matrix components.

## Discussion

Polyvalent TIA proteins have pleitropic effects on RNA biology and function as cell sensors to sustain homeostasis and facilitate adaptation, survival or death responses to a great variety of stressing conditions involving environmental and epigenetic challenges in the short- and long-term [19], [22]–[25], [28], [44]. To do this, these multifunctional regulators orchestrate transcriptome dynamics associated with transcriptional and/or post-transcriptional regulatory programs, which regulate and/or modulate molecular events and processes with relevant biological implications in cellular phenotypes and behaviours related with apoptosis, inflammation, viral infections, embryogenesis and oncogenesis [13]–[25]. Through genome-wide expression profiling approaches, we have identified many important genes/RNAs and have made a preliminary attempt to construct the regulatory pathways implicated in TIA-downregulated HeLa cells. Herein, we report that sustained TIA down-regulation has a functional impact on a human transcriptome associated to differentially-expressed genes and RNA isoforms generated by alternative splicing and/or promoter usage which are prevalently linked to gene categories related with focal/cell adhesion, extracellular matrix, membrane and cytoskeleton dynamics.

Cancer cells often exhibit hyperactive signalling pathways activated by membrane components. Cell and focal adhesions and cytoskeleton dynamics provide contact between neighbouring cells or between a cell and the extracellular matrix and, as such, play essential roles in the regulation of migration, proliferation, differentiation and apoptosis, and also tumorigenesis [45]. The reduction of TIA expression results in altered expression of extracellular matrix, membrane and cytoskeleton components and also signal transduction genes [23], [24], [44], [46], thus providing an additional molecular basis for the observation that sustained TIA down-regulation contributes to the deleterious phenotypes observed in HeLa cells lacking TIA proteins [23]–[25]. Notably, the down-signature enriched category included the focal and cell adhesion components (as for example, ALCAM, AMBP, CD9, DMKN, FLRT2, FOLR1, ITGA6, L1CAM, NOTCH3, OLR1, PCDHA1, PPP2R5C, RSPO3, TNNC1 and TSPAN8). These components play important roles in cell biology, and phenotypes linked to transformed cancer cells [45], [47], [48]. Moreover, genes in the more significantly up-regulated network module included collagens (as for example, COL1A1, COL1A2, COL3A1 and COL8A1) and extracellular matrix-related components (as for example, CD36, CFH, CGA, FBN2, IGFBP7, LGALS8, LOX, PDGFC and TNC). These observations strengthen the association of this gene subset with most transformed cell phenotypes and relate it to cell growth and survival responses [47], [48]. The effects of TIA expression on extracellular matrix and focal/cell adhesion phenomena would depend on how transcriptome dynamics are integrated in a given cell type and physiological context, and could have significant implications in inflammatory diseases and cancer progression [17], [19], [22]–[25], [49], [50].

TIA proteins are able to bind the same sequence motifs and sites on some human RNAs [12]–[16], and they can regulate and/or modulate some overlapping aspects of the human transcriptome [12]–[16]. A previous TIA-iCLIP analysis in HeLa cells showed that the highest enrichment density of cDNAs were seen in introns and 3′-UTRs [12]. These observations are consistent with the role of TIA proteins in pre-mRNA splicing, localization, stability and/or translational regulation, via binding U-, C- and AU-rich sequence elements located at the 5′ spliced sites and the 5′ and 3′-UTRs, respectively [9]–[21]. It is interesting to note that no overlap was observed between gene targets reported to be regulated by TIA1 at the translational level [13], and genes predicted *in silico* to be regulated by TIA1/TIAR at the alternative pre-mRNA splicing level [51]. This observation suggests that TIA proteins could be regulating and/or modulating distinct subsets of genes at the splicing and translational levels. The TIA-iCLIP data have expanded the total number of post-transcriptional events and associated gene functions that could be predicted to be regulated by TIA proteins. Thus, the estimated frequency of cellular events regulated by TIA1/TIAR via specific-sequences motifs might be approximately 20–30% [3], [4], [12], [51].

Tumorigenesis is caused both by genetic and epigenetic events. The progressive acquisition of mutations in oncogenes or tumor-suppressor genes might act in concert with epigenetic events, such as functional down-regulation of TIA proteins, to give cells a competitive growth advantage. TIA1 and TIAR genes are mutated [52] and down-regulated [18], [21], [25], [44], [49], [50] in several types of human cancers. In human transformed cells, TIA proteins regulate the transcription, alternative splicing, stability and/or translation of many target genes associated with tumor development and progression, involving the control of cell proliferation, apoptosis, angiogenesis, invasion, metastasis, evasion of immune recognition and metabolic reprogramming [13]–[25]. Thus, the sustained reduction of TIA proteins could facilitate the acquisition of oncogenic phenotypes [25] characterized by severe alterations in cell proliferation/growth, invasion and morphology, via modulation of several gene expression layers involving changes to global and specific translational rates of cell-cycle G2/M phase transition and DNA replication/repair factors encoding mRNAs [24] as well as actin [23] and tubulin [46] cytoskeletons, together with transcriptome dynamics linked to extracellular matrix, focal/cell adhesion and membrane regulatory events. Thus, prolonged TIA knockdown may lead to cell proliferation and neoplastic growth by simultaneously activating translation of mRNAs that encode proteins involved in cell cycle progression and cell dynamics. These findings also suggest cooperation of transcriptomic and translational programs that underpin prevalent biological effects on TIA-associated gene expression networks. This cellular and molecular scenario could contribute to the different phenotypes observed in TIA-knocked down HeLa cells. Therefore, our observations support the idea that these proteins might function as cell growth and tumor suppressor genes, through the regulation and/or modulation of many RNAs which are targeted by transcriptional and post-transcriptional regulatory events. However, further investigation is required to discern the cellular and molecular mechanisms underlying TIA protein control/modulation of gene expression. Results from these studies could potentially give insights into the differential gene networks that contribute to cell phenotypes observed in the absence of these regulators, both at short- and long-term, in homeostasis and environmental stress conditions.

## Supporting Information

Figure S1 **shRNA-mediated knockdown of TIA1 and TIAR in HeLa cells.** HeLa cells silenced for expression of TIA1, TIAR or HuR were stained with anti-TIA1, anti-TIAR, anti-HuR and anti-α-tubulin antibodies and were visualized by confocal microscopy, as described [23], [24]. The scale bar is 10 µm.
(PDF)

Figure S2 **Metrical data of massive-scale poly(A+) RNA sequencing analysis.**
(XLS)

Figure S3 **Summary of contig distribution on human chromosomes in control, TIA1 and TIAR-silenced HeLa cells.** The RNA-seq read density along the lengths of the human chromosomes is illustrated. Each bar represents the log_2_ of the frequency reads plotted against chromosome coordinates. RNA-seq data were mapped to the UCSC Human genome build 19. The RNA map corresponding to RNA binding proteins TIA1 and TIAR is included at the bottom. The results were adapted using the TIA1 and TIAR *in vivo* ultraviolet (UV)-crosslinking and immunoprecipitation (iCLIP) analysis provided by the Ule laboratory [12].
(PDF)

Figure S4 **List of massive sequencing-predicted genes in TIA1-silenced HeLa cells. Functional clusters based on Gene Ontology (GO) and Kyoto Encyclopedia of Genes and Genomes (KEGG) databases.**
(XLS)

Figure S5 **List of massive sequencing-predicted genes in TIAR-silenced HeLa cells. Functional clusters based on GO and KEGG databases.**
(XLS)

Figure S6 **List of shared and contrasting expressed genes and their GO/KEGG analysis from TIA1 and TIAR-silenced HeLa cells.**
(XLS)

Figure S7 **List of differentially expressed genes in TIA-silenced HeLa cells by microarray analysis. Functional clusters based on GO and KEGG databases.**
(XLS)

Figure S8 **List of primer pair sequences used in QPCR analysis.**
(XLS)

Figure S9 **Effect of TIA1 and TIAR knockdown on cell adhesion.** Cell adhesion of control and TIA-knocked down HeLa cells was assessed using either plastic, BSA-coated or collagen-coated plates. Cells were seeded and processed as indicated. Thereafter, the number of adhered cells was quantified by measuring the conversion of MTT into DMSO-soluble formazan by living cells, at 570 nm. The represented values were normalized and expressed relative to control values (whose value was fixed arbitrarily to 1 and are mean ± standard error of the mean (SEM) of at least two independent experiments.
(PDF)

Figure S10 **Effect of TIA1 and/or TIAR knockdown on transcriptional activation of the COL1A2 gene promoter.** Schematic representation of the COL1A2 human gene promoter is shown. *Cis*-acting consensus sequences are represented by boxes. Control and TIA1 and/or TIAR-silenced HeLa cells were transiently cotransfected with the COL1A2 promoter-driven firefly luciferase construct together with a GFP-expressing plasmid (used as a transfection control). The represented values –the ratio between luciferase relative light units (RLU)/GFP expression measured by Western blot– were normalized and expressed relative to the control sample, whose value was fixed arbitrarily to 1, and are mean ± SEM of at least two independent experiments.
(PDF)

## References

[1] Tian Q, Streuli M, Saito H, Schlossman SF, Anderson P (1991) A polyadenylate binding protein localized to the granules of cytolytic lymphocytes induces DNA fragmentation in target cells. Cell 67: 629–639. 1934064 10.1016/0092-8674(91)90536-8

[2] Kawakami A, Tian Q, Duan X, Streuli M, Schlossman SF, et al (1992) Identification and functional characterization of a TIA-1-related nucleolysin. Proc Natl Acad Sci 89: 8681–8685. 1326761 10.1073/pnas.89.18.8681 PMC49984

[3] Barbosa-Morais NL, Irimia M, Pan Q, Xiong HY, Gueroussov S, et al (2012) The evolutionary landscape of alternative splicing in vertebrate species. Science 338: 1587–1593. 23258890 10.1126/science.1230612

[4] Merkin J, Russell C, Chen P, Burge CB (2012) Evolutionary dynamics of gene and isoform regulation in mammalian tissues. Science 338: 1593–1599. 23258891 10.1126/science.1228186 PMC3568499

[5] Suswam EA, Li YY, Mahtani H, King PH (2005) Novel DNA-binding properties of the RNA-binding protein TIAR. Nucleic Acids Res 33: 4507–4518. 16091628 10.1093/nar/gki763 PMC1184220

[6] McAlinden A, Liang L, Mukudai Y, Imamura T, Sandell LJ (2007) Nuclear protein TIA1 regulates COL2A1 alternative splicing and interacts with precursor mRNA and genomic DNA. J Biol Chem 282: 24444–24454. 17580305 10.1074/jbc.M702717200

[7] Das R, Yu J, Zhang Z, Gygi MP, Krainer AR, et al (2007) SR proteins function in coupling RNAP II transcription to pre-mRNA splicing. Mol Cell 26: 867–881. 17588520 10.1016/j.molcel.2007.05.036

[8] Kim HS, Wilce MC, Yoga YM, Pendini NR, Gunzburg MJ, et al (2011) Different modes of interaction by TIAR and HuR with target RNA and DNA. Nucleic Acids Res 39: 1117–1130. 21233170 10.1093/nar/gkq837 PMC3035456

[9] Del Gatto-Konczak F, Bourgeois CF, Le Guiner C, Kister L, Gesnel MC, et al (2000) The RNA-binding protein TIA1 is a novel mammalian splicing regulator acting through intron sequences adjacent to a 5′ splice site. Mol Cell Biol 20: 6287–6299. 10938105 10.1128/mcb.20.17.6287-6299.2000 PMC86103

[10] Förch P, Puig O, Kedersha N, Martínez C, Granneman S, et al (2000) The apoptosis-promoting factor TIA1 is a regulator of alternative pre-mRNA splicing. Mol Cell 6: 1089–1098. 11106748 10.1016/s1097-2765(00)00107-6

[11] Izquierdo JM, Majós N, Bonnal S, Martínez C, Castelo R, et al (2005) Regulation of Fas alternative splicing by antagonistic effects of TIA1 and PTB on exon definition. Mol Cell 19: 475–484. 16109372 10.1016/j.molcel.2005.06.015

[12] Wang Z, Kayikci M, Briese M, Zarnack K, Luscombe NM, et al (2010) iCLIP predicts the dual splicing effects of TIA-RNA interactions. PLoS Biol 8: e1000530. 21048981 10.1371/journal.pbio.1000530 PMC2964331

[13] López de Silanes I, Galbán S, Martindale JL, Yang X, Mazan-Mamczarz K, et al (2005) Identification and functional outcome of mRNAs associated with RNA-binding protein TIA1. Mol Cell Biol 25: 9520–9531. 16227602 10.1128/MCB.25.21.9520-9531.2005 PMC1265820

[14] Mazan-Mamczarz K, Lal A, Martindale JL, Kawai T, Gorospe M (2006) Translational repression by RNA-binding protein TIAR. Mol Cell Biol 26: 2716–2727. 16537914 10.1128/MCB.26.7.2716-2727.2006 PMC1430315

[15] Kim HS, Kuwano Y, Zhan M, Pullmann R Jr, Mazan-Mamczarz K, et al (2007) Elucidation of a C-rich signature motif in target mRNAs of RNA-binding protein TIAR. Mol Cell Biol 27: 6806–6817. 17682065 10.1128/MCB.01036-07 PMC2099219

[16] Yamasaki S, Stoecklin G, Kedersha N, Simarro M, Anderson P (2007) T-cell intracellular antigen-1 (TIA1)-induced translational silencing promotes the decay of selected mRNAs. J Biol Chem 282: 30070–30077. 17711853 10.1074/jbc.M706273200

[17] Piecyk M, Wax S, Beck AR, Kedersha N, Gupta M, et al (2000) TIA1 is a translational silencer that selectively regulates the expression of TNF-alpha. EMBO J 19: 4154–4163. 10921895 10.1093/emboj/19.15.4154 PMC306595

[18] Liao B, Hu Y, Brewer G (2007) Competitive binding of AUF1 and TIAR to MYC mRNA controls its translation. Nat Struct Mol Biol 14: 511–518. 17486099 10.1038/nsmb1249

[19] Reyes R, Alcalde J, Izquierdo JM (2009) Depletion of T-cell intracellular antigen (TIA)-proteins promotes cell proliferation. Genome Biol 10: R87. 19709424 10.1186/gb-2009-10-8-r87 PMC2745768

[20] Damgaard CK, Lykke-Andersen J (2011) Translational coregulation of 5′TOP mRNAs by TIA-1 and TIAR. Genes Dev 25: 2057–2068. 21979918 10.1101/gad.17355911 PMC3197204

[21] Gottschald OR, Malec V, Krasteva G, Hasan D, Kamlah F, et al (2010) TIAR and TIA1 mRNA-binding proteins co-aggregate under conditions of rapid oxygen decline and extreme hypoxia and suppress the HIF-1α pathway. J Mol Cell Biol 2: 345–356. 20980400 10.1093/jmcb/mjq032

[22] Sánchez-Jiménez C, Carrascoso I, Barrero J, Izquierdo JM (2013) Identification of a set of miRNAs differentially expressed in transiently TIA-depleted HeLa cells by genome-wide profiling. BMC Mol Biol 14: 4. 23387986 10.1186/1471-2199-14-4 PMC3600012

[23] Carrascoso I, Sánchez-Jiménez C, Izquierdo JM (2014) Long-term reduction of T-cell intracellular antigens leads to increased beta-actin expression. Mol Cancer 13: 90. 24766723 10.1186/1476-4598-13-90 PMC4113145

[24] Carrascoso I, Sánchez-Jiménez C, Izquierdo JM (2014) Genome-wide profiling reveals a role for T-cell intracellular antigens TIA1 and TIAR in the control of translational specificity in HeLa cells. Biochem J 461: 43–50. 24927121 10.1042/BJ20140227

[25] Izquierdo JM, Alcalde J, Carrascoso I, Reyes R, Ludeña MD (2011) Knockdown of T-cell intracellular antigens triggers cell proliferation, invasión and tumor growth. Biochem J 435: 337–344. 21284605 10.1042/BJ20101030

[26] Beck AR, Miller JJ, Anderson P, Streuli M (1998) RNA-binding protein TIAR is essential for primordial germ cell development. Proc Natl Acad Sci 95: 2331–2336. 9482885 10.1073/pnas.95.5.2331 PMC19335

[27] Kharraz Y, Salmand PA, Camus A, Auriol J, Gueydan C, et al (2010) Impaired embryonic development in mice overexpressing the RNA-binding protein TIAR. PLoS One 5: e11352. 20596534 10.1371/journal.pone.0011352 PMC2893167

[28] Sánchez-Jiménez C, Izquierdo JM (2013) T-cell intracellular antigen (TIA)-proteins deficiency in murine embryonic fibroblats alters cell cycle progression and induces autophagy. PLoS One 8: e75127. 24086455 10.1371/journal.pone.0075127 PMC3782481

[29] Twine NA, Janitz K, Wilkins MR, Janitz M (2011) Whole transcriptome sequencing reveals gene expression and splicing differences in brain regions affected by Alzheimer’s disease. PLoS One 6: e16266. 21283692 10.1371/journal.pone.0016266 PMC3025006

[30] Trapnell C, Pachter L, Salzberg SL (2009) TopHat: discovering splice junctions with RNA-seq. Bioinformatics 25: 1105–1111. 19289445 10.1093/bioinformatics/btp120 PMC2672628

[31] Trapnell C, Williams BA, Pertea G, Mortazavi A, Kwan G, et al (2010) Transcript assembly and quantification by RNA-seq reveals unannotated transcripts and isoform switching during cell differentiation. Nat Biotechnology 28: 511–515. 10.1038/nbt.1621 PMC3146043 20436464

[32] Jiang H, Wong WH (2009) Statistical inferences for isoform expression in RNA-seq. Bioinformatics 25: 1026–1032. 19244387 10.1093/bioinformatics/btp113 PMC2666817

[33] Zweig AS, Karolchik D, Kuhn RM, Haussler D, Kent WJ (2008) UCSC genome browser tutorial. Genomics 92: 75–84. 18514479 10.1016/j.ygeno.2008.02.003

[34] Langmead B, Trapnell C, Pop M, Salzberg SL (2009) Ultrafast and memory- efficient alignment of short DNA sequences to the human genome. Genome Biol 10: R25. 19261174 10.1186/gb-2009-10-3-r25 PMC2690996

[35] Smyth GK, Speed TP (2003) Normalization of cDNA microarray data. Methods 31: 265–273. 14597310 10.1016/s1046-2023(03)00155-5

[36] Bolstad BM, Irizarry RA, Astrand M, Speed TP (2003) A comparison of normalization methods for high density oligonucleotide array data based on bias and variance. Bioinformatic 19: 185–193. 10.1093/bioinformatics/19.2.185 12538238

[37] Smyth GK (2001) Limma: linear models for microarray data. In: Gentleman R, Carey V, Dudoit S, Irizarry R, Huber W, editors. Bioinformatics and Computational Biology Solutions using R and Bioconductor. Springer: New York. 397–420.

[38] Benjamini Y, Drai D, Elmer G, Kafkafi N, Golani I (2001) Controlling the false discovery rate in behavior genetics research. Behav Brain Res 125: 279–284. 11682119 10.1016/s0166-4328(01)00297-2

[39] Oliveros JC (2007) FIESTA@BioinfoGP.An interactive server for analyzing DNA microarray experiments with replicates. FIESTA Viewer New FIESTA Server Available: http://bioinfogp.cnb.csic.es/tools/FIESTA.

[40] Carmona-Saez P, Chagoyen M, Tirado F, Carazo JM, Pascual-Montano A (2007) GENECODIS: a web-based tool for finding significant concurrent annotations in gene lists. Genome Biol 8: R3. 17204154 10.1186/gb-2007-8-1-r3 PMC1839127

[41] Nogales-Cadenas R, Carmona-Saez P, Vazquez M, Vicente C, Yang X, et al (2009) GeneCodis: interpreting gene lists through enrichment analysis and integration of diverse biological information. Nucleic Acids Res 37: W317–322. 19465387 10.1093/nar/gkp416 PMC2703901

[42] Chen Y (2011) Cell adhesion assay. Bio-protocol 1: e98.

[43] Miyazaki H, Kobayashi R, Ishikawa H, Awano N, Yamagoe S, Miyazaki Y, Matsumoto T (2012) Activation of COL1A2 promoter in human fibroblasts by Escherichia coli. FEMS Immunol Med Microbiol 65: 481–487. 22534007 10.1111/j.1574-695X.2012.00979.x

[44] Heck MV, Azizov M, Stehning T, Walter M, Kedersha N, et al (2014) Dysregulated expression of lipid storage and membrane dynamics factors in Tia1 knockout mouse nervous tissue. Neurogenetics 15: 135–144. 24659297 10.1007/s10048-014-0397-x PMC3994287

[45] Watt FM, Huck WT (2013) Role of the extracellular matrix in regulating stem cell fate. Nat Rev Mol Cell Biol 14: 467–473. 23839578 10.1038/nrm3620

[46] Li X, Rayman JB, Kandel ER, Derkatch IL (2014) Functional Role of Tia1/Pub1 and Sup35 Prion Domains: Directing Protein Synthesis Machinery to the Tubulin Cytoskeleton. Mol Cell 55: 305–318. 24981173 10.1016/j.molcel.2014.05.027 PMC4425694

[47] Duperret EK, Ridky TW (2013) Focal adhesion complex proteins in epidermis and squamous cell carcinoma. Cell Cycle 12: 3272–3285. 24036537 10.4161/cc.26385 PMC3885638

[48] González DM, Medici D (2014) Signaling mechanisms of the epithelial-mesenchymal transition. Sci Signal 7: re8. 25249658 10.1126/scisignal.2005189 PMC4372086

[49] Karalok HM, Karalok E, Saglam O, Torun A, Guzeloglu-Kayisli O, et al (2014) mRNA-binding Protein TIA-1 Reduces Cytokine Expression in Human Endometrial Stromal Cells and is Down-Regulated in Ectopic Endometrium. J Clin Endocrinol Metab Aug 20: jc20133488. 10.1210/jc.2013-3488 PMC4255110 25140393

[50] Hamdollah Zadeh MA, Amin EM, Hoareau-Aveilla C, Domingo E, Symonds KE, et al (2014) Alternative splicing of TIA-1 in human colon cancer regulates VEGF isoform expression, angiogenesis, tumour growth and bevacizumab resistance. Mol Oncol Aug 20 pii: S1574-7891(14)00172-0. doi:10.1016/j.molonc.2014.07.017 10.1016/j.molonc.2014.07.017 PMC4286123 25224594

[51] Aznarez I, Barash Y, Shai O, He D, Zielenski J, et al (2008) A systematic analysis of intronic sequences downstream of 5′ splice sites reveals a widespread role for U-rich motifs and TIA1/TIAL1 proteins in alternative splicing regulation. Genome Res 18: 1247–1258. 18456862 10.1101/gr.073155.107 PMC2493427

[52] Gonzalez-Pérez A, Pérez-Llamas C, Deu-Pons J, Tamborero D, Schroeder MP, et al (2013) IntOGen-mutations identifies cancer drivers across tumor types. Nat Methods 10: 1081–1082. 24037244 10.1038/nmeth.2642 PMC5758042

