# Supplementary figures and images for: Long-Term Reduction of T-Cell Intracellular Antigens Reveals a Transcriptome Associated with Extracellular Matrix and Cell Adhesion Components
Source: PLoS One. 2014 Nov 18;9(11):e113141. doi: 10.1371/journal.pone.0113141 (PMC4236147; doi:10.1371/journal.pone.0113141)

Figure S1

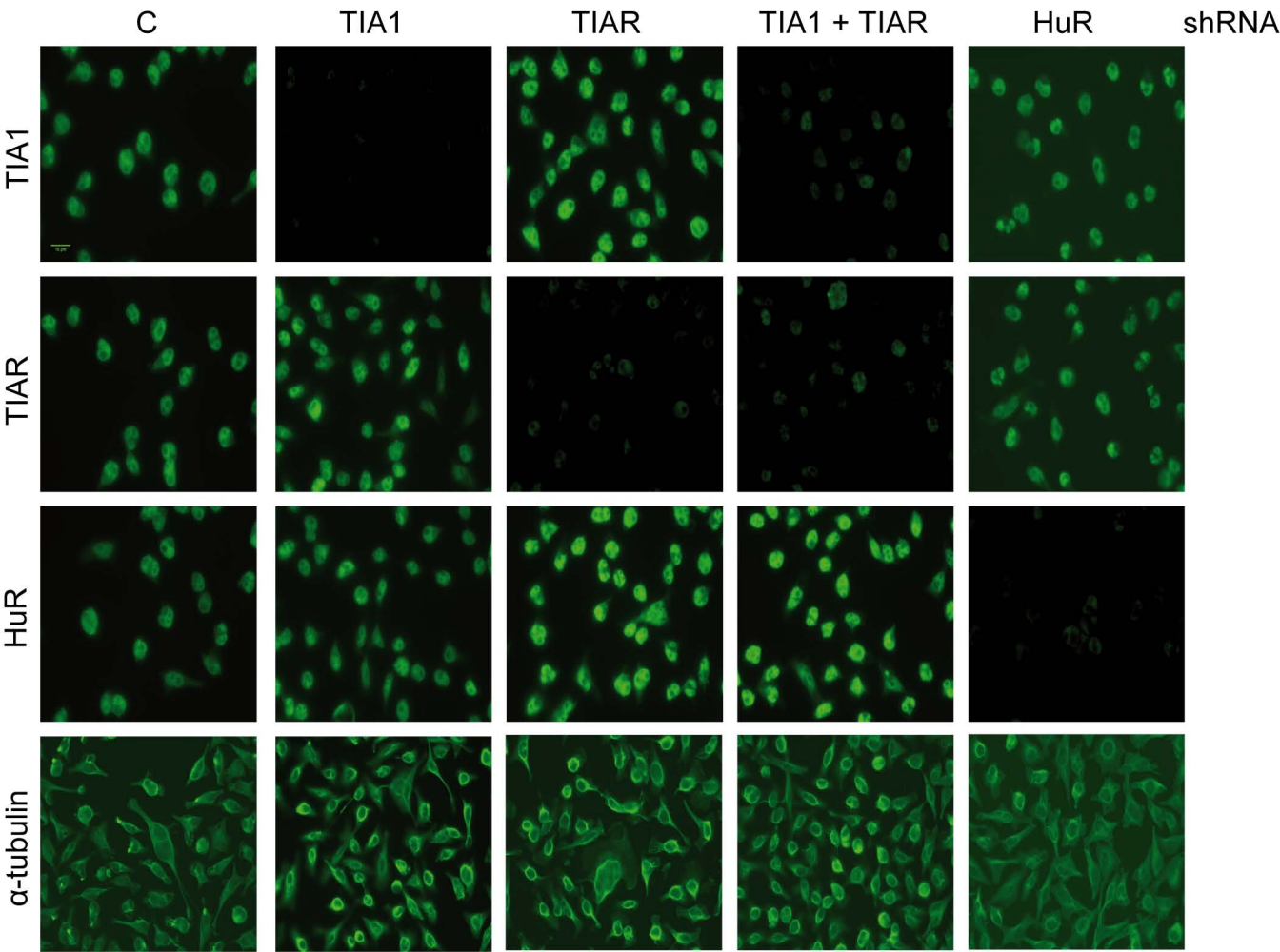

Supplement: Figure S1 — shRNA-mediated knockdown of TIA1 and TIAR in HeLa cells. HeLa cells silenced for expression of TIA1, TIAR or HuR were stained with anti-TIA1, anti-TIAR, anti-HuR and anti-α-tubulin antibodies and were visualized by confocal microscopy, as described [23], [24]. The scale bar is 10 µm. (PDF) [file pone.0113141.s001.pdf]

Figure S3

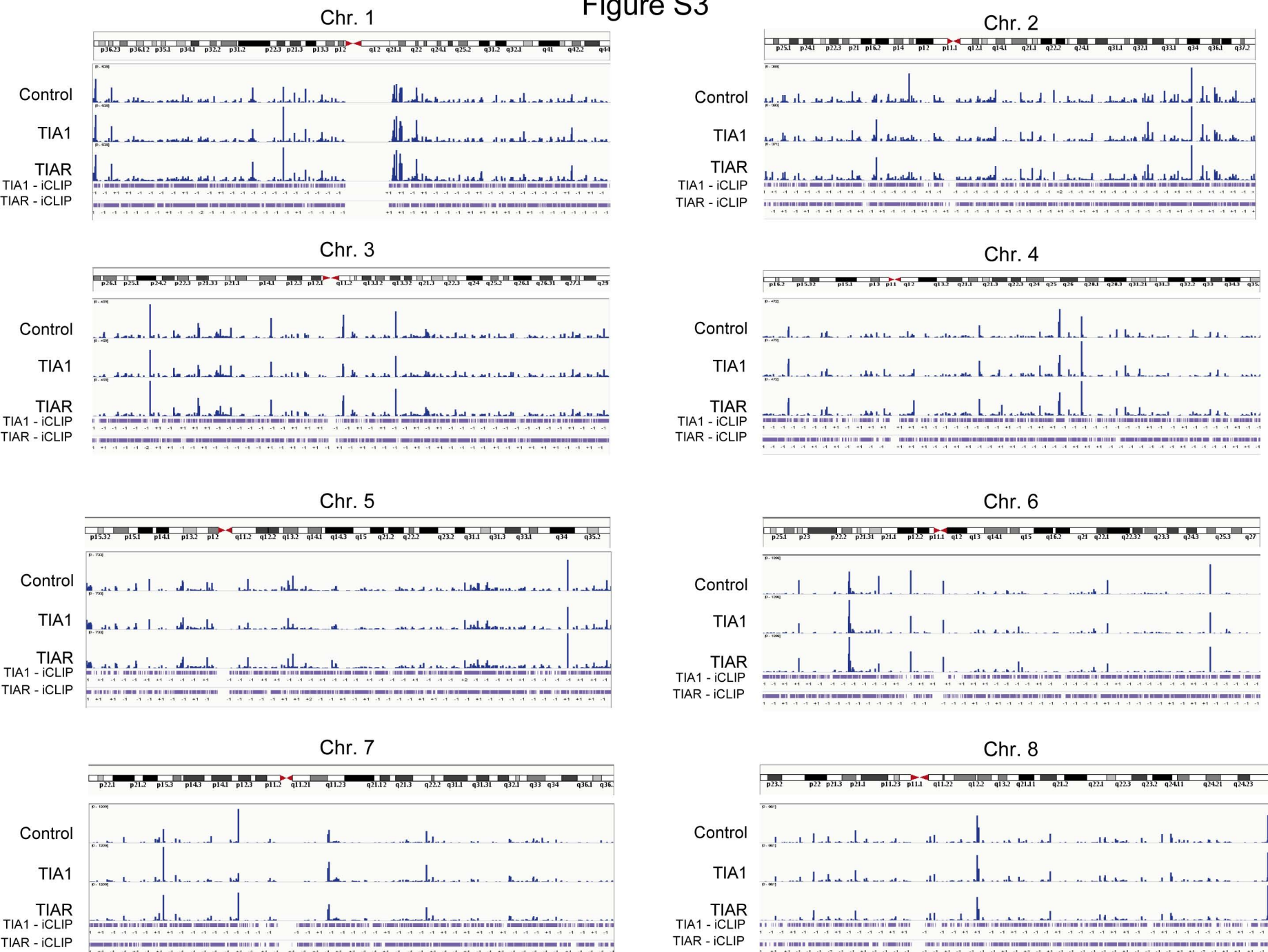

Figure S3

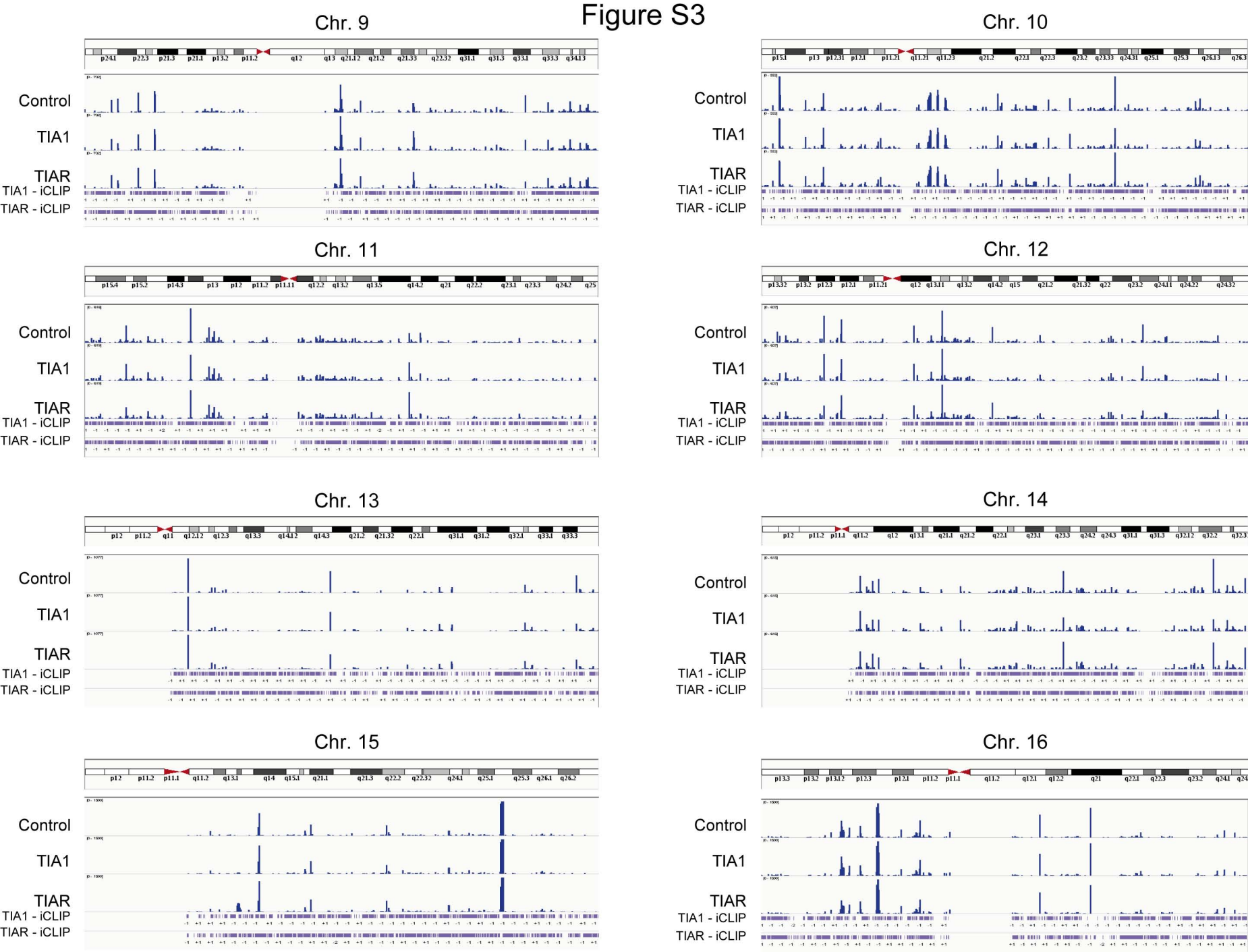

Figure S3

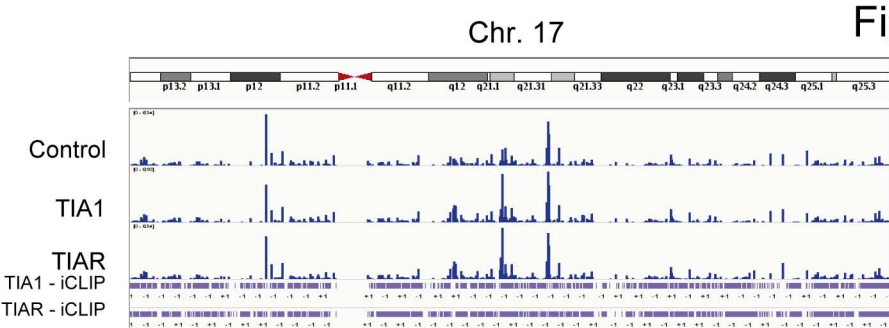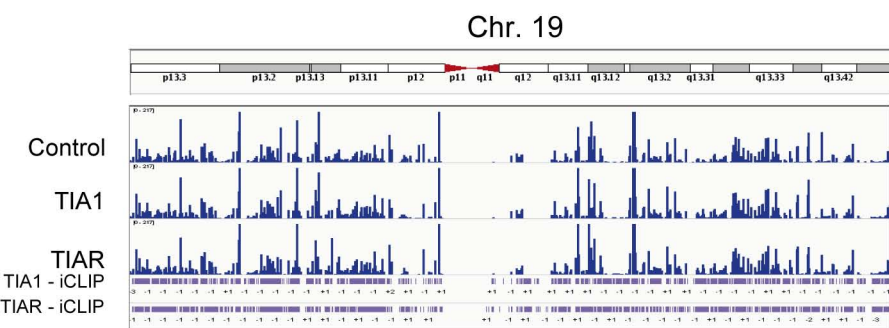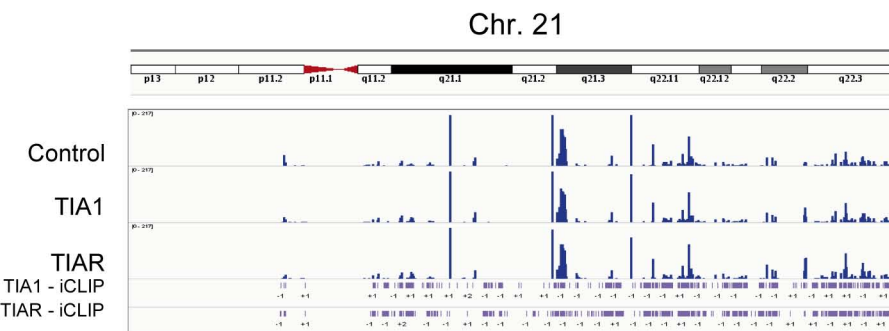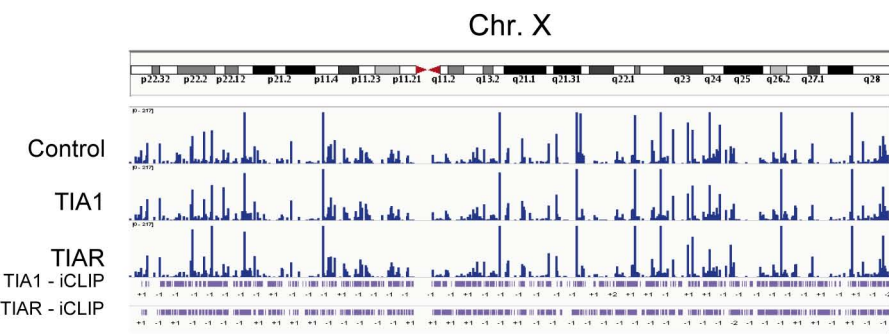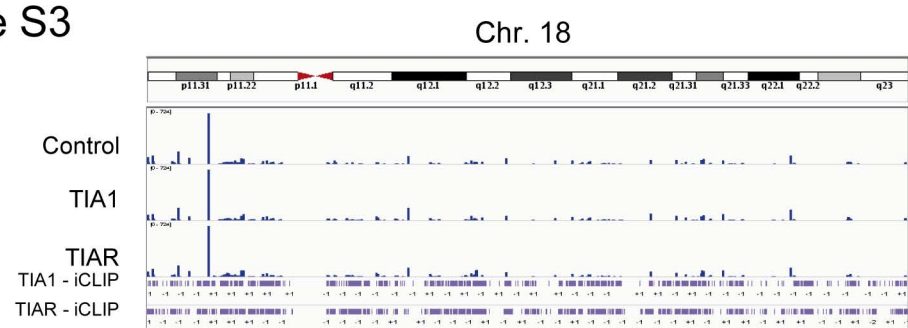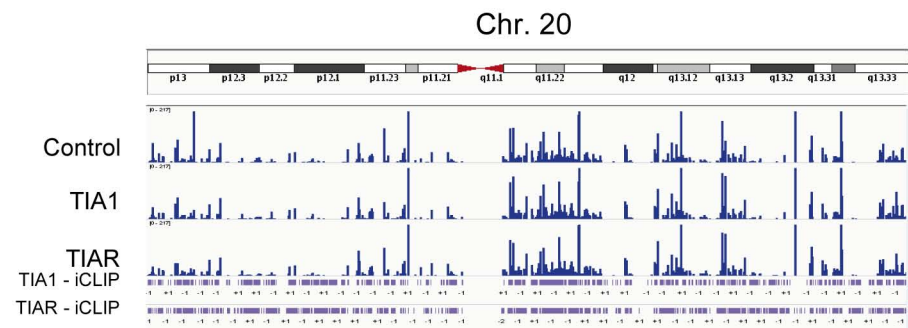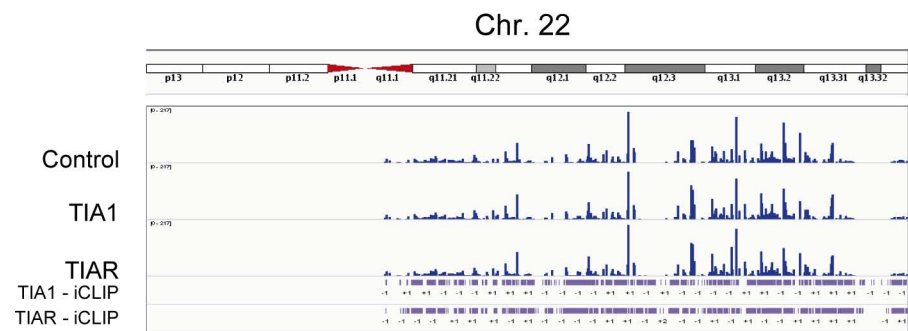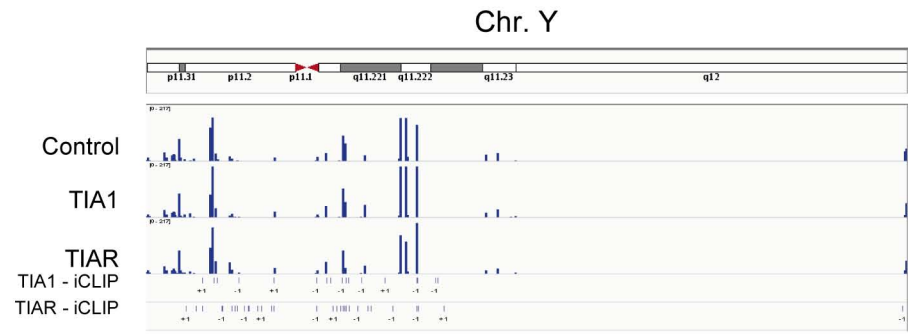

Supplement: Figure S3 — Summary of contig distribution on human chromosomes in control, TIA1 and TIAR-silenced HeLa cells. The RNA-seq read density along the lengths of the human chromosomes is illustrated. Each bar represents the log2 of the frequency reads plotted against chromosome coordinates. RNA-seq data were mapped to the UCSC Human genome build 19. The RNA map corresponding to RNA binding proteins TIA1 and TIAR is included at the bottom. The results were adapted using the TIA1 and TIAR in vivo ultraviolet (UV)-crosslinking and immunoprecipitation (iCLIP) analysis provided by the Ule laboratory [12]. (PDF) [file pone.0113141.s003.pdf]

Figure S9

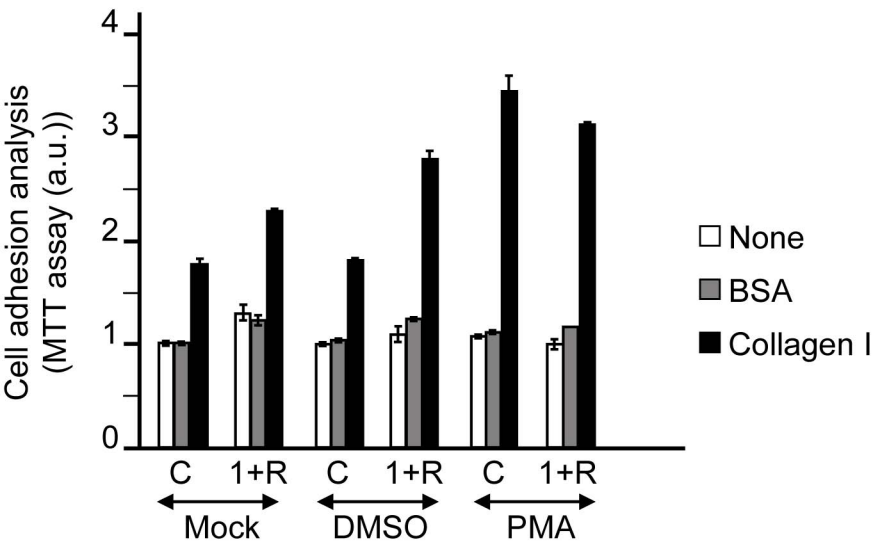

Supplement: Figure S9 — Effect of TIA1 and TIAR knockdown on cell adhesion. Cell adhesion of control and TIA-knocked down HeLa cells was assessed using either plastic, BSA-coated or collagen-coated plates. Cells were seeded and processed as indicated. Thereafter, the number of adhered cells was quantified by measuring the conversion of MTT into DMSO-soluble formazan by living cells, at 570 nm. The represented values were normalized and expressed relative to control values (whose value was fixed arbitrarily to 1 and are mean ± standard error of the mean (SEM) of at least two independent experiments. (PDF) [file pone.0113141.s009.pdf]

Figure S10

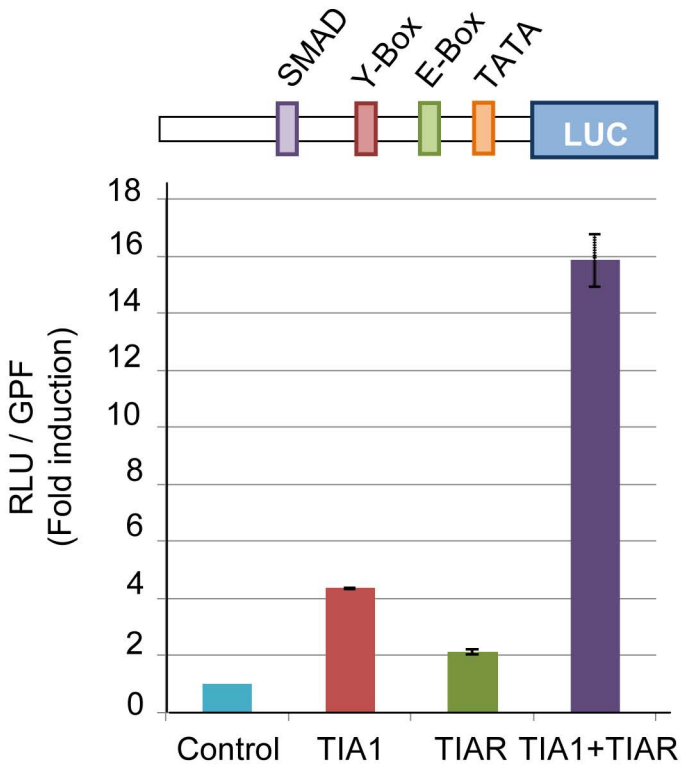

Supplement: Figure S10 — Effect of TIA1 and/or TIAR knockdown on transcriptional activation of the COL1A2 gene promoter. Schematic representation of the COL1A2 human gene promoter is shown. Cis-acting consensus sequences are represented by boxes. Control and TIA1 and/or TIAR-silenced HeLa cells were transiently cotransfected with the COL1A2 promoter-driven firefly luciferase construct together with a GFP-expressing plasmid (used as a transfection control). The represented values –the ratio between luciferase relative light units (RLU)/GFP expression measured by Western blot– were normalized and expressed relative to the control sample, whose value was fixed arbitrarily to 1, and are mean ± SEM of at least two independent experiments. (PDF) [file pone.0113141.s010.pdf]
